# Supplementary material for: Seroprevalence of anti-SARS-CoV-2 antibodies in women attending antenatal care in eastern Ethiopia: a facility-based surveillance
Source: BMJ Open. 2021 Nov 24;11(11):e055834. doi: 10.1136/bmjopen-2021-055834 (PMC8613670; doi:10.1136/bmjopen-2021-055834)
Supplement: Supplementary data [file bmjopen-2021-055834supp001.pdf]

Supplementary table 1: Characteristics of women who were in the surveillance in the two study areas between April 2020 and March 2021 and sampled at random for the study.

Characteristics	Tested		Not tested		Total
	n	%	n	%	N
All	1,447	43.7	1,865	56.3	3,312
Age in years					
14-19	192	44.3	241	55.7	433
20-24	588	42.5	797	57.6	1385
25-29	400	41.8	556	58.2	956
30-34	210	49.0	219	51.1	529
>=35	56	51.9	52	48.2	108
Residence					
Urban	984	43.5	1,277	56.5	2,261
Rural	461	44.2	582	55.8	1,043
Number of children					
None	552	43.6	713	56.4	1,265
1-5	823	43.4	1,073	56.6	1,896
6-10	61	46.2	71	53.8	132
Trimester of visit					
First	366	39.6	558	60.4	924
Second	658	45.0	805	55.0	1,463
Third	423	45.7	502	54.3	925
Comorbidities					
None	1,439	43.7	1,857	56.3	3,296
At least one*	8	50.0	8	50.0	16
COVID symptoms†					
No	1,389	43.7	1,791	56.3	3,180
Yes	51	42.9	68	57.1	119
Month of sampling					
April 2020	144	38.0	235	62.0	379
May 2020	144	32.4	301	67.6	445
June 2020	143	37.9	234	62.1	377
July 2020	80	69.0	36	31.0	116
August 2020	80	37.4	134	62.6	214
September 2020	80	41.7	112	58.3	192
October 2020	72	100.0	0	0.0	72
November 2020	80	44.7	99	55.3	179
December 2020	160	53.9	137	46.1	297
January 2021	160	55.9	126	44.1	286
February 2021	160	55.0	131	45.0	291
March 2021	144	31.0	320	69.0	464

* chronic liver, renal, cardiovascular or 'other' disease

† at least one of cough, fever, headache or difficulty breathing
