# Supplementary material for: Seroprevalence of anti-SARS-CoV-2 antibodies in women attending antenatal care in eastern Ethiopia: a facility-based surveillance
Source: BMJ Open. 2021 Nov 24;11(11):e055834. doi: 10.1136/bmjopen-2021-055834 (PMC8613670; doi:10.1136/bmjopen-2021-055834)
Supplement: Author's manuscript [file bmjopen-2021-055834.draft_revisions.pdf]

BMJ Open

BMJ Open is committed to open peer review. As part of this commitment we make the peer review history of every article we publish publicly available.

When an article is published we post the peer reviewers' comments and the authors' responses online. We also post the versions of the paper that were used during peer review. These are the versions that the peer review comments apply to.

The versions of the paper that follow are the versions that were submitted during the peer review process. They are not the versions of record or the final published versions. They should not be cited or distributed as the published version of this manuscript.

BMJ Open is an open access journal and the full, final, typeset and author-corrected version of record of the manuscript is available on our site with no access controls, subscription charges or pay-per-view fees (http://bmjopen.bmj.com).

If you have any questions on BMJ Open's open peer review process please email info.bmjopen@bmj.com

BMJ Open

Seroprevalence of anti–SARS-CoV-2 antibodies in women attending antenatal care in eastern Ethiopia

Journal:	*BMJ Open*
Manuscript ID	bmjopen-2021-055834
Article Type:	Original research
Date Submitted by the Author:	25-Jul-2021
Complete List of Authors:	Assefa, Nega; Haramaya University; London School of Hygiene & Tropical Medicine<br>Regassa, Lemma; Haramaya University<br>Teklemariam, Zelalem; Haramaya University<br>Oundo, Joseph; London School of Hygiene & Tropical Medicine<br>Madrid-Castillo, Lola; London School of Hygiene & Tropical Medicine<br>Dessie, Yadeta; Haramaya University<br>Scott, A; London School of Hygiene and Tropical Medicine,
Keywords:	COVID-19, Health policy < HEALTH SERVICES ADMINISTRATION & MANAGEMENT, Protocols & guidelines < HEALTH SERVICES ADMINISTRATION & MANAGEMENT, Infection control < INFECTIOUS DISEASES, Prenatal diagnosis < OBSTETRICS

SCHOLARONE™
Manuscripts

I, the Submitting Author has the right to grant and does grant on behalf of all authors of the Work (as defined in the below author licence), an exclusive licence and/or a non-exclusive licence for contributions from authors who are: i) UK Crown employees; ii) where BMJ has agreed a CC-BY licence shall apply, and/or iii) in accordance with the terms applicable for US Federal Government officers or employees acting as part of their official duties; on a worldwide, perpetual, irrevocable, royalty-free basis to BMJ Publishing Group Ltd ("BMJ") its licensees and where the relevant Journal is co-owned by BMJ to the co-owners of the Journal, to publish the Work in this journal and any other BMJ products and to exploit all rights, as set out in our licence.

The Submitting Author accepts and understands that any supply made under these terms is made by BMJ to the Submitting Author unless you are acting as an employee on behalf of your employer or a postgraduate student of an affiliated institution which is paying any applicable article publishing charge ("APC") for Open Access articles. Where the Submitting Author wishes to make the Work available on an Open Access basis (and intends to pay the relevant APC), the terms of reuse of such Open Access shall be governed by a Creative Commons licence – details of these licences and which Creative Commons licence will apply to this Work are set out in our licence referred to above.

Other than as permitted in any relevant BMJ Author's Self Archiving Policies, I confirm this Work has not been accepted for publication elsewhere, is not being considered for publication elsewhere and does not duplicate material already published. I confirm all authors consent to publication of this Work and authorise the granting of this licence.

For peer review only

Seroprevalence of anti–SARS-CoV-2 antibodies in women attending antenatal care in eastern Ethiopia

Nega Assefa[1,2, *], Lemma Demissie Regassa[1], Zelalem Teklemarriam[1], Joseph Oundo[2], Lola Madrid[2], Yadeta Dessie[1], J. Anthony G. Scott[2]

[1] College of Health and Medical Sciences, Haramaya University, Ethiopia

[2] London School of Hygiene & Tropical Medicine, UK

* Corresponding author
Nega Assefa
Email: negaassefa@yahoo.com

Contact address
NA: negaassefa@yahoo.com
LDR: esraeldemiss@gmail.com
ZT: zelalemtmariam@yahoo.com
LM: lola.madrid-castillo@lshtm.ac.uk
JO: joseph.oundo@lshtm.ac.uk
YD: yad_de2005@yahoo.com
JAGS: anthony.scott@lshtm.ac.uk

Target Journal

Word counts

Abstract	245
Main text	2489

For peer review only

Abstract

Objective: We conducted serosurveillance of anti–SARS-CoV-2 antibodies among pregnant women attending their first antenatal care.

Setting: The surveillance was set in one referral hospital in Harar, one district hospital and one health centre located in Haramaya district in rural eastern Ethiopia.

Participants: We collected questionnaire data and a blood sample from 3,312 pregnant women between April 1, 2020 and March 31, 2021. We selected 1,447 blood samples at random and assayed these for anti-SARS-CoV-2 antibodies at Hararghe Health Research laboratory using WANTAI® SARS-CoV-2 Rapid Test for total immunoglobulin.

Outcome: We assayed for anti-SARS-CoV-2 antibodies and temporal trends in seroprevalence were analysed with a χ^2 test for trend and multivariable binomial regression.

Results: Among 1,447 sera tested, 83 were positive for anti–SARS-CoV-2 antibodies giving a crude seroprevalence of 5.7% (95% CI 4.6%, 7.0%). Of 160 samples tested in April-May, 2020, none was seropositive; the first seropositive sample was identified in June and seroprevalence rose steadily thereafter (χ^2 test for trend, p=0.003) reaching a peak of 11.8% in February, 2021. In the multivariable model, seroprevalence was approximately 3% higher in first-trimester mothers compared to later presentations, and rose by 0.75% (95% CI 0.31%, 1.20%) per month of calendar time.

Conclusions: This clinical convenience sample illustrates the dynamic of the SARS-CoV-2 epidemic in young adults in eastern Ethiopia; infection was rare before June 2020 but it spread in a linear fashion thereafter, rather than following intermittent waves, and reached 10% by the beginning of 2021. After one year of surveillance, most pregnant mothers remained susceptible.

For peer review only

Keywords: COVID 19, COVID-19 seroprevalence, COVID-19 among pregnant women, COVID-19 in Ethiopia

Key summaries:

What is already known about this subject?

- Information on the cumulative incidence of SARS-CoV-2 in is scarce and little is known about.

What are the new findings?"

- The crude seroprevalence anti–SARS-CoV-2 antibodies among first time ANC attendants was 5.7%.
- The first seropositive sample was identified in June 2020 and seroprevalence rose steadily thereafter reached a peak of 11.8% in February, 2021.
- Seroprevalence was approximately 3% higher in first-trimester mothers compared to later presentations, and rose by 0.75% per month of calendar time.

How might it impact on clinical practice in the foreseeable future?

- The disease spreads among asymptomatic community members unnoticed.
- A healthy looking could be source of infection for susceptible individuals. This will eventually create pressure on health facilities to treat symptomatic cases.
- The effect of COVID infection on birth outcomes needs to be investigated.

Strengths and limitations

Strength

- Selecting healthy looking first time ANC attendants to represent the general population
- Instituting a surveillance in both tertiary and primary health facilities located in urban and rural areas represents the urban and rural community.

Limitation

- Its focus on women visited the health care facilities and missing several not attending health care.

Introduction

In Ethiopia, the first case of COVID-19 was reported on 13 March 2020. By the end of March 2021 there were 206,589 reports of COVID-19 infection and 2,865 coronavirus-related deaths. In a country with an estimated population, in 2019, of 112 million this represents a cumulative incidence of SARS-CoV-2 infection of only 0.2% after a full year of transmission. Many cases of COVID-19 present with mild symptoms and, in Ethiopia, three quarters of PCR-positive cases have no symptoms [1,2]. Access to PCR testing in Ethiopia is also sparse. Monitoring the epidemic by detecting symptomatic cases is, therefore, highly insensitive. In these circumstances, seroprevalence of anti-SARS-CoV-2 antibodies can provide a more accurate estimator of cumulative incidence. Undertaking community serosampling during the pandemic is difficult when travel and household access are constrained by control measures. Expectant mothers, however, are likely to continue to seek health services throughout the pandemic and they can be used as a continuously-available proxy population to estimate the cumulative incidence among young adults [3-5]. In addition, serological surveillance is simple to implement at ANC clinic visits because anti-SARS-CoV-2 antibodies can be assayed in the residual blood volumes of routine samples collected for clinical screening for anemia and maternal infectious diseases.

Planning and provision of health care during a major epidemic like COVID-19 pose substantial logistical and clinical challenges. Information on the shape of the epidemic curve is critical to inform public health responses. The dynamics of seroprevalence reflect the epidemic curve and can provide an estimate of the effective reproduction number. Seroprevalence also indicates the likelihood of approaching transmission control through population immunity. This study aimed to assess the trend in seroprevalence of anti-SARS-CoV-2 antibodies throughout the first year of the

For peer review only - http://bmjopen.bmj.com/site/about/guidelines.xhtml

epidemic by assaying anti-SARS-CoV-2 antibodies among pregnant women attending ante-natal clinic at three different health facilities in the area around Harar, eastern Ethiopia.

Material and methods

Study area and period

The surveillance was conducted between April 1, 2020 and March 31, 2021 at Awoday Health Centre and Haramaya District Hospital, both in Haramaya District, and in Hiwot Fana Specialized Referral University Hospital in Harar. Hiwot Fana is the largest referral and teaching hospital in eastern Ethiopia and receives tertiary referrals from Harari region, East Oromia, Somali region, and Dire Dawa City. It is one of the ten regional centres designated by the Federal Ministry of Health to manage the COVID-19 epidemic. Haramaya Hospital was rapidly designated a COVID-19 treatment facility and women seeking ANC services were therefore referred to Awoday Health Centre after April 16, 2020.

Study design, population and sample size

At the end of March 2020, we integrated Health facility-based surveillance into the routine clinical care of pregnant women at Hiwot Fana Hospital, Awoday Health Centre and Haramaya Hospital. The study population comprised 3306 pregnant women attending their first antenatal care in these three facilities during the surveillance period. A total 78 women were excluded because they were not willing to provide blood sample. Routine antenatal care includes serological screening for HIV, syphilis, and toxoplasma infection during pregnancy undertaken in two blood samples; the first blood sample is taken at 16 weeks' gestation or at the first ANC visit, if later.

For peer review only

Socio-demographic data and information on pregnancy, clinical symptoms of COVID-19 and co-morbidities was collected by trained nurses. COVID-19 symptoms were defined as at least one of cough, fever, headache or difficulty breathing. Data quality and completeness were checked daily.

Laboratory analyses

For the anti–SARS-CoV-2 antibodies test, residual blood samples from the routine ANC tests were transferred to a test-tube containing clot activator by trained medical laboratory technologists working in each health facility. The blood samples were allowed to clot and serum was separated by centrifugation at 3000 RPM for 10 minutes. Serum samples were stored at 2-8°C at each site and transported in cool boxes to Hararghe Health Research Laboratory where they were stored at -80°C.

Samples were tested using WANTAI® SARS-CoV-2 Ab Rapid Test. The test is a lateral flow assay in a cassette format designed for the qualitative detection of total antibodies to SARS-CoV-2 in human serum. The receptor-binding domain of the SARS-CoV-2 spike protein is bound at the Test Zone (T) and antibodies are bound at the Control Zone (C) of the cassette. The test has a sensitivity of 100% and specificity of 98.8% under validation performed by the manufacturer; independent validation of the test found a sensitivity of 89% [6]. All the stored serum samples, tests reagents and cassettes were brought to room temperature (15-30°C) thirty minutes before performing the test; 10µl of serum specimen and two drops of diluent buffer were added into the specimen window. Results were read and interpreted as reactive/positive (Red line on C and T) or non-reactive/negative (Red line on C) after 15-20 minutes [6,7]. Serum samples taken ≥14 days after a positive PCR test from COVID-19 infected individuals were used as quality control.

For peer review only

Patient and Public Involvement statement

In COVID pandemic, much attention have been given for COVID symptomatic cases, while asymptomatic cases are either not addressed or neglected. In this study, it has been discussed with public health authorities and community representatives in the study area in several occasions. Women have much interaction in the community as well in the family. They represent the community better than other person to learn the dynamics of a disease of such kind. For this reason, a surveillance was set among pregnant women attending ANC in local health facilities. These women come to health facilities for check-up, and investigators agreed that, first time ANC visitors are best representatives.

As clients were visiting the antenatal clinic, nurses at the clinic explained the issue of COVID and asked women if she would like to participate by giving blood sample for testing. Pregnant women were encouraged to raise any unclear points and then referred to the laboratory for blood sample collection as they do other tests.

Data from this surveillance is made available in public repository. Aggregated data has been shred to authorities in districts and health facilities to plan for actions against the problem. Pregnant women attending ANC were given information on the disease nationally, regionally and locally, and they were also given health education on COVID prevention.

Statistical analysis

We used STATA version 16.0 for statistical analysis. We estimated unadjusted seroprevalence of SARS-CoV-2 IgG antibody with a 95% confidence interval (CI). We did not make adjustment for the test performance characteristics because the manufacturer's validation assay, found very high sensitivity and specificity. We examined the univariate association between individual

For peer review only

characteristics and seropositivity using χ^2 and multivariable associations using binomial regression. The trend in seropositivity with time was tested with a χ^2 test for trend and in the multivariable model.

Ethical consideration

The study was confined to residual clinical blood sample testing and anonymized questionnaire data. It was conducted as part of a public health surveillance, with the approval of the director of each of the three health facilities and the data were made available to relevant bodies including the Regional Health Bureau (Harari and Oromia) and the Ethiopian Public Health Institute (EPHI). Ethical clearance was secured from Institutional Health Research Ethical Review Committee of the College of Health and Medical Sciences, Haramaya University, Ethiopia with clearance number 123/2021.

Results

Demographic characteristics of the study participants

Between April 1, 2020 and March 31, 2021 there were 3,313 first visits to the antenatal clinics; 1,532 (46.24%) at Hiwot Fana Hospital, 1781 (53.75%) at Awoday Health Centre and Haramaya Hospital. At these, we interviewed and collected blood samples from 3,312 women. We tested a random sample of 1,447 blood specimens (Table 1); 752 (52%) were from Haramaya District (Awoday Health Centre and Haramaya Hospital) and 695 (48%) were from Hiwot Fana Hospital; 984 (68%) were urban residents.

Among the population sample tested, the mean (SD) age was 23.9 (4.7) years and ages ranged from 15 to 45 years. The mean (SD) number of children per mother was 1.5 (1.8). The median

For peer review only

(IQR) gestational age at the first antenatal visit was 20 (13-28) weeks. Only 51 (3.5%) had COVID-19 symptoms at the time of sampling and 8 (<1%) had a history of comorbidity, given as chronic liver, renal, cardiovascular or 'other' disease. Respiratory diseases, chronic neurological disease, diabetes mellitus, and cancer were not reported by any participant.

Seroprevalence of SARS-CoV-2 antibodies

Of 1,447 samples tested, 83 (5.7%, 95% CI 4.6, 7.0%) were positive for anti-SARS-CoV-2 antibodies. The first seropositive sample was identified on June 11, 2020, and seroprevalence rose progressively thereafter, with the exception of March 2021, where it dropped sharply (χ^2 for trend for the whole year, p=0.003; Figure 1).

Seroprevalence also varied significantly by trimester of pregnancy and co-morbidity but not by clinic, residence or COVID-19 symptoms (Table 2). Given the linear growth in seroprevalence (Figure 1) and better model fit based on Bayesian information criterion, we modelled prevalence associations as risk differences rather than risk ratios. In a multivariable binomial regression model, the prevalence difference was -3.2% (95% CI -6.7, -0.4%) and -3.0% (95% CI -6.8,-0.8%) among women in their second and third trimesters, respectively, compared with those in the first trimester and the prevalence difference was 0.75% (95% CI 0.31, 1.20%) per month of calendar time.

Discussion

The study provides a simple description of the dynamic of SARS-CoV-2 epidemic in an area where reliable data are extremely rare. In a population of attendees at ante-natal clinics in three sites in eastern Ethiopia, antibodies against SARS-CoV-2 first appeared in June 2020 and seroprevalence rose steadily month on month reaching approximately 10% at the beginning of 2021. Although the point estimate for March 2021 is substantially lower, the data as a whole evince a strong linear

For peer review only

trend and this single estimate is most likely to have deviated from the general direction by chance. If these results are reliable, they indicate that the epidemic is progressing here at a considerably lower rate than in other settings in East Africa and that the greater majority of the population remains uninfected, suggesting that the epidemic is still at an early stage.

The principal limitations of the study are the potential generalisability of the population under surveillance and the validity of the serological assay employed. Pregnant women have been used as an indicator population in prior pandemics, including HIV [8], but also for SARS-CoV-2, both in high-income settings [4,5,9-13] and low- and middle-income settings, including in neighbouring Kenya [3]. The principal advantage of sampling pregnant women is that they remain one of the few patient groups for whom health services cannot be postponed until after the pandemic has passed. They are permitted and encouraged to attend even in the face of social and movement restrictions, and so provide a consistent and reliable sampling group. The principal limitation of this group is their restriction on age and sex, however, in most settings, including other East African countries, seroprevalence does not vary significantly by sex and the cumulative incidence in women is likely to represent the infection history of both sexes [14]. Similarly, in most settings young adults are the group most likely to be infected by SARS-CoV-2 and so the seroprevalence estimates here are likely to represent the highest risk in the whole population; other age groups, particularly children and the elderly, are likely to have lower seroprevalence [15,16].

The World Health Organization has deprecated the use of rapid tests for SARS-CoV-2 antibodies for individual diagnosis but recognises their potential value in research [17]. WHO has also recommended and endorsed quantitative analysis of IgG antibodies using ELISA and has distributed the WANTAI ELISA kit to countries undertaking serosurveillance. Reliance on ELISA, however, limits the range of settings in which serosurveillance can be undertaken and

For peer review only

lateral flow tests have been successfully employed for recurrent community-based nationwide surveys in the UK [18]. When seroprevalence is low, as at the beginning of our study, an assay with imperfect specificity may detect more false positives than true positives. The specificity of the WANTAI rapid test has been estimated by the manufacturer at 98.8%. We assayed 80 samples each month in April and May 2020 without observing a single positive test, suggesting that the specificity is indeed very high. Even if the positive results identified in June included false positives, the progressive rise in seropositivity with time is most unlikely to be influenced materially by a small fraction of false positive results.

The assay sensitivity may also be imperfect in detecting prior infection because the assay was originally calibrated against sera from symptomatic cases, who generally have higher antibody levels than asymptomatic individuals [19], and because pregnant women who were infected several months ago may have experienced waning of antibody levels and seroreversion [20-24]. In general, seroreversion is less problematic in assays that measure total immunoglobulin and in those that target spike antigens, compared to nucleocapsid antigens [22,25], so problem of waning in this study is unlikely to be substantial. Furthermore, if sensitivity is unlikely to decline over time, imperfect sensitivity would not affect the shape of the rising seroprevalence line, though it would underestimate the gradient. If, as estimated in one validation study, the WANTAI rapid test has a sensitivity of only 89% [6], adjustment for test-performance characteristics would elevate our reported seroprevalence results by a factor of 1.12.

The results are in contrast to most other settings studied which record a sharp take-off in seroprevalence once transmission begins, often rising quickly to high levels. In Kenya for example, women attending an ANC clinic in Kilifi had seroprevalence of 0%, 2% and 11% in consecutive months September-November 2021; those attending ANC clinic in Nairobi had a seroprevalence

For peer review only

of 50% in August 2020 [3]. The pattern illustrated in eastern Ethiopia is more indicative of a gradually spreading epidemic curve suggesting an effective reproduction number much closer to 1.

In Juba, South Sudan, seroprevalence was 22% in a household survey in August-September 2020 [26]; in Kenya, a national estimate for seroprevalence, based on testing blood transfusion donors, was 4.3% in May 2020 [14] and 9.1% two months later [27]. Health Care Workers in Nairobi, Kenya, had a seroprevalence of 44% in August 2020; those in two rural hospitals had seroprevalence of 12-13% in November 2020 [28]. Finally, in Addis Ababa seroprevalence, estimated in May 2020, was 3.0% [29]. Although all these studies used different laboratory assays and varied statistical adjustments, collectively, they suggest that transmission in eastern Ethiopia began later than in much of the rest of the region, including the state capital, and has progressed more slowly.

Conclusion

In summary if seroprevalence is a reliable indicator of cumulative incidence, SARS-CoV-2 infection is spreading slowly but steadily in eastern Ethiopia. This contrasts sharply with the recurrent waves of PCR-positive infections apparent in the national surveillance system. One year after the start of the epidemic approximately 10% of women attending ante-natal clinics are seropositive implying that the COVID-19 epidemic is still at an early stage in eastern Ethiopia.

For peer review only

References

1 Abdela, S. G. *et al.* Clinical Profile and Treatment of COVID-19 Patients: Experiences from an Ethiopian Treatment Center. *Am J Trop Med Hyg*, doi:10.4269/ajtmh.20-1356 (2020).

2 Abraha, H. E. *et al.* Clinical features and risk factors associated with morbidity and mortality among patients with COVID-19 in northern Ethiopia. *Int J Infect Dis* **105**, 776-783, doi:10.1016/j.ijid.2021.03.037 (2021).

3 Lucinde, R. *et al.* Sero-surveillance for IgG to SARS-CoV-2 at antenatal care clinics in two Kenyan referral hospital. *medRxiv* **submitted** (2021).

4 Lumley, S. F. *et al.* SARS-CoV-2 antibody prevalence, titres and neutralising activity in an antenatal cohort, United Kingdom, 14 April to 15 June 2020. *Euro Surveill* **25**, doi:10.2807/1560-7917.ES.2020.25.41.2001721 (2020).

5 Villalaín, C. *et al.* Seroprevalence analysis of SARS-CoV-2 in pregnant women along the first pandemic outbreak and perinatal outcome. *PLOS ONE* **15**, e0243029, doi:10.1371/journal.pone.0243029 (2020).

6 Weidner, L. *et al.* Quantification of SARS-CoV-2 antibodies with eight commercially available immunoassays. *Journal of Clinical Virology* **129**, 104540, doi:https://doi.org/10.1016/j.jcv.2020.104540 (2020).

7 The WANTAI SARS-CoV-2 Ab Rapid Test. *Beijing Wantai Biological Pharmacy Enterprise Co., Ltd. No.31* <www.ystwt.com > (2020).

8 Kigadye, R. M. *et al.* Sentinel surveillance for HIV-1 among pregnant women in a developing country: 3 years' experience and comparison with a population serosurvey. *AIDS* **7**, 849-855, doi:10.1097/00002030-199306000-00014 (1993).

For peer review only

9 Cosma, S. *et al.* The "scar" of a pandemic: Cumulative incidence of COVID-19 during the first trimester of pregnancy. *J Med Virol* **93**, 537-540, doi:10.1002/jmv.26267 (2021).

10 Crovetto, F. *et al.* Seroprevalence and presentation of SARS-CoV-2 in pregnancy. *Lancet* **396**, 530-531, doi:10.1016/S0140-6736(20)31714-1 (2020).

11 Flannery, D. D. *et al.* SARS-CoV-2 seroprevalence among parturient women in Philadelphia. *Sci Immunol* **5**, doi:10.1126/sciimmunol.abd5709 (2020).

12 Haizler-Cohen, L., Davidov, A., Blitz, M. J. & Fruhman, G. Severe acute respiratory syndrome coronavirus 2 antibodies in pregnant women admitted to labor and delivery units. *Am J Obstet Gynecol* **224**, 112-114, doi:10.1016/j.ajog.2020.09.022 (2021).

13 Mattern, J. *et al.* Post lockdown COVID-19 seroprevalence and circulation at the time of delivery, France. *PLoS One* **15**, e0240782, doi:10.1371/journal.pone.0240782 (2020).

14 Uyoga, S. *et al.* Seroprevalence of anti-SARS-CoV-2 IgG antibodies in Kenyan blood donors. *Science* **371**, 79-82, doi:10.1126/science.abe1916 (2021).

15 Murhekar, M. V. *et al.* SARS-CoV-2 antibody seroprevalence in India, August-September, 2020: findings from the second nationwide household serosurvey. *Lancet Glob Health* **9**, e257-e266, doi:10.1016/S2214-109X(20)30544-1 (2021).

16 Pollan, M. *et al.* Prevalence of SARS-CoV-2 in Spain (ENE-COVID): a nationwide, population-based seroepidemiological study. *Lancet* **396**, 535-544, doi:10.1016/S0140-6736(20)31483-5 (2020).

17 World Health Organization. https://www.who.int/news-room/commentaries/detail/advice-on-the-use-of-point-of-care-immunodiagnostic-tests-for-covid-19 (2020).

For peer review only

18 Moshe, M. *et al.* SARS-CoV-2 lateral flow assays for possible use in national covid-19 seroprevalence surveys (React 2): diagnostic accuracy study. *BMJ* **372**, n423, doi:10.1136/bmj.n423 (2021).

19 Takahashi, S., Greenhouse, B. & Rodriguez-Barraquer, I. Are Seroprevalence Estimates for Severe Acute Respiratory Syndrome Coronavirus 2 Biased? *J Infect Dis* **222**, 1772-1775, doi:10.1093/infdis/jiaa523 (2020).

20 Ibarrondo, F. J. *et al.* Rapid Decay of Anti-SARS-CoV-2 Antibodies in Persons with Mild Covid-19. *N Engl J Med* **383**, 1085-1087, doi:10.1056/NEJMc2025179 (2020).

21 Long, Q. X. *et al.* Clinical and immunological assessment of asymptomatic SARS-CoV-2 infections. *Nat Med* **26**, 1200-1204, doi:10.1038/s41591-020-0965-6 (2020).

22 Lumley, S. F. *et al.* The duration, dynamics and determinants of SARS-CoV-2 antibody responses in individual healthcare workers. *Clin Infect Dis*, doi:10.1093/cid/ciab004 (2021).

23 Muecksch, F. *et al.* Longitudinal Serological Analysis and Neutralizing Antibody Levels in Coronavirus Disease 2019 Convalescent Patients. *J Infect Dis* **223**, 389-398, doi:10.1093/infdis/jiaa659 (2021).

24 Ward, H. *et al.* Prevalence of antibody positivity to SARS-CoV-2 following the first peak of infection in England: Serial cross-sectional studies of 365,000 adults. *Lancet Reg Health Eur* **4**, 100098, doi:10.1016/j.lanepe.2021.100098 (2021).

25 Post, N. *et al.* Antibody response to SARS-CoV-2 infection in humans: A systematic review. *PLoS One* **15**, e0244126, doi:10.1371/journal.pone.0244126 (2020).

26 Wiens, K. E. *et al.* Seroprevalence of anti-SARS-CoV-2 IgG antibodies in Juba, South Sudan: a population-based study. *medRxiv*, doi:10.1101/2021.03.08.21253009 (2021).

For peer review only

27 Adetifa, I. M. O. *et al.* Temporal trends of SARS-CoV-2 seroprevalence in transfusion blood donors during the first wave of the COVID-19 epidemic in Kenya. *medRxiv*, doi:https://www.medrxiv.org/content/10.1101/2021.02.09.21251404v1 (2021).

28 Etyang, A. O. *et al.* Seroprevalence of Antibodies to SARS-CoV-2 among Health Care Workers in Kenya. *Clin Infect Dis*, doi:10.1093/cid/ciab346 (2021).

29 Kempen, J. H. *et al.* SARS-CoV-2 Serosurvey in Addis Ababa, Ethiopia. *Am J Trop Med Hyg* **103**, 2022-2023, doi:10.4269/ajtmh.20-0816 (2020).

For peer review only

Author contribution

NA and JAGS lead overall Surveillance. NA, JAGS, ZT and LDR develop concept of the Surveillance, analyzed the data and wrote the manuscript. ZT provided microbiome data analysis, and interpretation. NA, JAGS, ZT, LDR, LM, JO and YD reviewed the manuscript and give critical feedback. All authors approved the submission of the manuscript to the journal.

Competing of interest

The authors declare no competing interests.

Funding:

Activities of the surveillance and Laboratory analysis was supported by Emory University as part of the CHAMPS network (www.champshealth.org). JAGS was funded by The Wellcome Trust through a Senior Research Fellowship (214320).

Data availability

Data is available in the following link and can be requested using the form in the link. https://dataverse.harvard.edu/dataset.xhtml?persistentId=doi:10.7910/DVN/XIWCXN.

Acknowledgements

Investigators would like to thank authorities of the health facilities for facilitating the surveillance and pregnant women for voluntarily participating in the study.

For peer review only

Figure 1: Temporal trend of seroprevalence of anti–SARS-CoV-2 antibodies among pregnant women presenting for first antenatal care in 3 ANC facilities, eastern Ethiopia, between April 1, 2020 and March 31, 2021

For peer review only

Table 1. Characteristics of 1,447 pregnant mothers attending their first antenatal care at the two study areas between April 2020 and March 2021 and sampled at random for the study.

**Characteristics**	**Haramaya District**		**Hiwot Fana Hospital**		**Total**	
	**n**	**%**	**n**	**%**	**n**	**%**
Age in years						
14-19	98	13.0	74	13.5	192	13.3
20-24	341	45.4	268	35.6	588	40.7
25-29	181	24.1	219	31.6	400	27.7
30-34	110	14.6	100	14.4	210	14.5
>=35	22	2.9	35	4.9	56	3.9
Residence						
Urban	577	76.8	407	58.7	984	68.1
Rural	174	23.2	287	41.4	461	31.9
Number of children						
None	298	40.0	254	36.8	552	38.4
1-5	422	56.6	401	58.1	823	57.3
6-10	26	3.5	35	5.1	61	4.3
Trimester of visit						
First	223	29.7	143	20.6	366	25.3
Second	416	55.3	242	34.8	658	45.5
Third	113	15.0	310	44.6	423	29.2
Comorbidities						
None	750	99.7	689	99.1	1,439	99.5
At least one*	2	0.3	6	0.9	8	0.6
COVID symptoms†						
No	721	96.4	668	96.5	1,389	96.5
Yes	27	3.6	24	3.5	51	3.5

* chronic liver, renal, cardiovascular or 'other' disease

† at least one of cough, fever, headache or difficulty breathing

For peer review only

Table 2. Seroprevalence of Anti-SARS-CoV-2 antibodies by participant characteristics.

**Characteristics**	**Tested N**	**Sero-positive n**	**Sero-prevalence %**	**χ^2 test**
Age in years				
14-19	192	7	3.7	p=0.19
20-24	588	42	7.1	
25-29	400	24	6.0	
30-34	210	9	4.3	
>=35	56	1	1.8	
ANC clinic				
Hiwot Fana Hospital	695	35	5.0	p=0.26
Haramaya Hospital	19	0	0.0	
Awoday Health Centre	733	48	6.6	
Residence				
Urban	984	57	5.8	p=0.91
Rural	461	26	5.6	
Number of children				
None	520	32	5.8	P=0.370
1-5	778	45	5.5	
6-10	55	6	9.8	
Trimester of visit				
First	366	31	8.5	p=0.034
Second	658	32	4.9	
Third	423	20	4.8	
Comorbidities				
None	1,439	81	5.6	p=0.019
At least one*	8	2	25.0	
COVID symptoms†				
No	1,389	82	5.9	p=0.24
Yes	51	1	2.0	

* chronic liver, renal, cardiovascular or ‘other’ disease

† at least one of cough, fever, headache or difficulty breathing

For peer review only

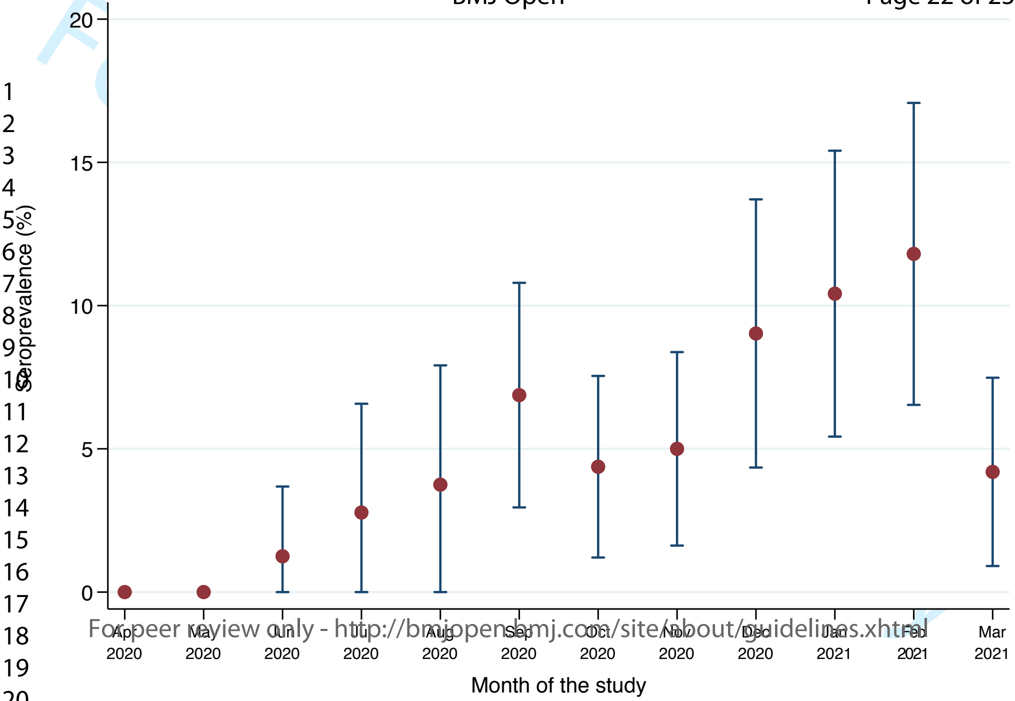

For peer review only - http://bmjopen.bmj.com/site/about/guidelines.xhtml

Seroprevalence of anti–SARS-CoV-2 antibodies in women attending antenatal care in eastern Ethiopia

STROBE Statement—Checklist of items that should be included in reports of ***cross-sectional studies***

	Item No	Recommendation	Page No
**Title and abstract**	1	(*a*) Indicate the study's design with a commonly used term in the title or the abstract	1
		(*b*) Provide in the abstract an informative and balanced summary of what was done and what was found	1
**Introduction**			
Background/rationale	2	Explain the scientific background and rationale for the investigation being reported	4
Objectives	3	State specific objectives, including any prespecified hypotheses	4-5
**Methods**			
Study design	4	Present key elements of study design early in the paper	5
Setting	5	Describe the setting, locations, and relevant dates, including periods of recruitment, exposure, follow-up, and data collection	5
Participants	6	(*a*) Give the eligibility criteria, and the sources and methods of selection of participants	5-6
Variables	7	Clearly define all outcomes, exposures, predictors, potential confounders, and effect modifiers. Give diagnostic criteria, if applicable	6
Data sources/ measurement	8*	For each variable of interest, give sources of data and details of methods of assessment (measurement). Describe comparability of assessment methods if there is more than one group	7
Bias	9	Describe any efforts to address potential sources of bias	7
Study size	10	Explain how the study size was arrived at	5
Quantitative variables	11	Explain how quantitative variables were handled in the analyses. If applicable, describe which groupings were chosen and why	6-7
Statistical methods	12	(*a*) Describe all statistical methods, including those used to control for confounding	7
		(*b*) Describe any methods used to examine subgroups and interactions	7
		(*c*) Explain how missing data were addressed	7
		(*d*) If applicable, describe analytical methods taking account of sampling strategy	7
		(*e*) Describe any sensitivity analyses	7
**Results**			
Participants	13*	(a) Report numbers of individuals at each stage of study—eg numbers potentially eligible, examined for eligibility, confirmed eligible, included in the study, completing follow-up, and analysed	8
		(b) Give reasons for non-participation at each stage	8
		(c) Consider use of a flow diagram	-
Descriptive data	14*	(a) Give characteristics of study participants (eg demographic, clinical, social) and information on exposures and potential confounders	8-10
		(b) Indicate number of participants with missing data for each variable of interest	8
Outcome data	15*	Report numbers of outcome events or summary measures	8-9

For peer review only - http://bmjopen.bmj.com/site/about/guidelines.xhtml

Seroprevalence of anti–SARS-CoV-2 antibodies in women attending antenatal care in eastern Ethiopia

Main results	16	(*a*) Give unadjusted estimates and, if applicable, confounder-adjusted estimates and their precision (eg, 95% confidence interval). Make clear which confounders were adjusted for and why they were included	8-9
		(*b*) Report category boundaries when continuous variables were categorized	9
		(*c*) If relevant, consider translating estimates of relative risk into absolute risk for a meaningful time period	-
Other analyses	17	Report other analyses done—eg analyses of subgroups and interactions, and sensitivity analyses	9
**Discussion**			
Key results	18	Summarise key results with reference to study objectives	9-10
Limitations	19	Discuss limitations of the study, taking into account sources of potential bias or imprecision. Discuss both direction and magnitude of any potential bias	10
Interpretation	20	Give a cautious overall interpretation of results considering objectives, limitations, multiplicity of analyses, results from similar studies, and other relevant evidence	10-12
Generalisability	21	Discuss the generalisability (external validity) of the study results	11-12
**Other information**			
Funding	22	Give the source of funding and the role of the funders for the present study and, if applicable, for the original study on which the present article is based	16

*Give information separately for exposed and unexposed groups.

Note: An Explanation and Elaboration article discusses each checklist item and gives methodological background and published examples of transparent reporting. The STROBE checklist is best used in conjunction with this article (freely available on the Web sites of PLoS Medicine at http://www.plosmedicine.org/, Annals of Internal Medicine at http://www.annals.org/, and Epidemiology at http://www.epidem.com/). Information on the STROBE Initiative is available at www.strobe-statement.org.

For peer review only

For peer review only - http://bmjopen.bmj.com/site/about/guidelines.xhtml

BMJ Open

Seroprevalence of anti–SARS-CoV-2 antibodies in women attending antenatal care in eastern Ethiopia: a facility-based surveillance

Journal:	*BMJ Open*
Manuscript ID	bmjopen-2021-055834.R1
Article Type:	Original research
Date Submitted by the Author:	08-Oct-2021
Complete List of Authors:	Assefa, Nega; Haramaya University; London School of Hygiene & Tropical Medicine<br>Regassa, Lemma; Haramaya University<br>Teklemariam, Zelalem; Haramaya University<br>Oundo, Joseph; London School of Hygiene & Tropical Medicine<br>Madrid-Castillo, Lola; London School of Hygiene & Tropical Medicine<br>Dessie, Yadeta; Haramaya University<br>Scott, A; London School of Hygiene and Tropical Medicine,
**Primary Subject Heading**:	Epidemiology
Secondary Subject Heading:	Global health, Public health, Infectious diseases
Keywords:	COVID-19, Health policy < HEALTH SERVICES ADMINISTRATION & MANAGEMENT, Protocols & guidelines < HEALTH SERVICES ADMINISTRATION & MANAGEMENT, Infection control < INFECTIOUS DISEASES, Prenatal diagnosis < OBSTETRICS

SCHOLARONE™
Manuscripts

I, the Submitting Author has the right to grant and does grant on behalf of all authors of the Work (as defined in the below author licence), an exclusive licence and/or a non-exclusive licence for contributions from authors who are: i) UK Crown employees; ii) where BMJ has agreed a CC-BY licence shall apply, and/or iii) in accordance with the terms applicable for US Federal Government officers or employees acting as part of their official duties; on a worldwide, perpetual, irrevocable, royalty-free basis to BMJ Publishing Group Ltd ("BMJ") its licensees and where the relevant Journal is co-owned by BMJ to the co-owners of the Journal, to publish the Work in this journal and any other BMJ products and to exploit all rights, as set out in our licence.

The Submitting Author accepts and understands that any supply made under these terms is made by BMJ to the Submitting Author unless you are acting as an employee on behalf of your employer or a postgraduate student of an affiliated institution which is paying any applicable article publishing charge ("APC") for Open Access articles. Where the Submitting Author wishes to make the Work available on an Open Access basis (and intends to pay the relevant APC), the terms of reuse of such Open Access shall be governed by a Creative Commons licence – details of these licences and which Creative Commons licence will apply to this Work are set out in our licence referred to above.

Other than as permitted in any relevant BMJ Author's Self Archiving Policies, I confirm this Work has not been accepted for publication elsewhere, is not being considered for publication elsewhere and does not duplicate material already published. I confirm all authors consent to publication of this Work and authorise the granting of this licence.

For peer review only

Seroprevalence of anti–SARS-CoV-2 antibodies in women attending antenatal care in eastern Ethiopia: a facility-based surveillance

Nega Assefa[1, 2, *], Lemma Demissie Regassa[1], Zelalem Teklemariam[1], Joseph Oundo[2], Lola Madrid[2], Yadeta Dessie[1], J. Anthony G. Scott[2]

[1] College of Health and Medical Sciences, Haramaya University, Ethiopia

[2] London School of Hygiene & Tropical Medicine, UK

* Corresponding author
Nega Assefa
Email: negaassefa@yahoo.com

Contact address
NA: negaassefa@yahoo.com
LDR: esraeldemiss@gmail.com
ZT: zelalemtmariam@yahoo.com
LM: lola.madrid-castillo@lshtm.ac.uk
JO: joseph.oundo@lshtm.ac.uk
YD: yad_de2005@yahoo.com
JAGS: anthony.scott@lshtm.ac.uk

Word counts

Abstract	245
Main text	2489

For peer review only

For peer review only - http://bmjopen.bmj.com/site/about/guidelines.xhtml

Abstract

Objective: We conducted serosurveillance of anti–SARS-CoV-2 antibodies among pregnant women attending their first antenatal care.

Setting: The surveillance was set in one referral hospital in Harar, one district hospital and one health centre located in Haramaya district in rural eastern Ethiopia.

Participants: We collected questionnaire data and a blood sample from 3,312 pregnant women between April 1, 2020 and March 31, 2021. We selected 1,447 blood samples at random and assayed these for anti-SARS-CoV-2 antibodies at Hararghe Health Research laboratory using WANTAI® SARS-CoV-2 Rapid Test for total immunoglobulin.

Outcome: We assayed for anti-SARS-CoV-2 antibodies and temporal trends in seroprevalence were analysed with a Chi square test for trend and multivariable binomial regression.

Results: Among 1,447 sera tested, 83 were positive for anti–SARS-CoV-2 antibodies giving a crude seroprevalence of 5.7% (95% CI 4.6%, 7.0%). Of 160 samples tested in April-May 2020, none was seropositive; the first seropositive sample was identified in June and seroprevalence rose steadily thereafter (Chi square test for trend, p=0.003) reaching a peak of 11.8% in February 2021. In the multivariable model, seroprevalence was approximately 3% higher in first-trimester mothers compared to later presentations, and rose by 0.75% (95% CI 0.31%, 1.20%) per month of calendar time.

Conclusions: This clinical convenience sample illustrates the dynamic of the SARS-CoV-2 epidemic in young adults in eastern Ethiopia; infection was rare before June 2020 but it spread in a linear fashion thereafter, rather than following intermittent waves, and reached 10% by the beginning of 2021. After one year of surveillance, most pregnant mothers remained susceptible.

For peer review only - http://bmjopen.bmj.com/site/about/guidelines.xhtml

Keywords: COVID 19, COVID-19 seroprevalence, COVID-19 among pregnant women, COVID-19 in Ethiopia

Strengths and limitations

- The surveillance was initiated quickly at the start of the pandemic and was pursued with consistent methods over a full calendar year
- Pregnant women are consistently available for surveillance throughout movement restrictions providing a practical and valid survey of seroprevalence trends
- Results from pregnant women may not be fully representative of older or younger women, nor of men at any age.
- SARS-CoV-2 antibodies were assayed using a lateral flow device which, though convenient, has inferior performance characteristics to ELISA

For peer review only
For peer review only - http://bmjopen.bmj.com/site/about/guidelines.xhtml

Introduction

In Ethiopia, the first case of COVID-19 was reported on 13 March 2020. By the end of March 2021, there were 206,589 reports of COVID-19 infection and 2,865 coronavirus-related deaths. In a country with an estimated population, in 2019, of 112 million this represents a cumulative incidence of SARS-CoV-2 infection of only 0.2% after a full year of transmission. Many cases of COVID-19 present with mild symptoms and, in Ethiopia, three quarters of PCR-positive cases have no symptoms [1 2]. Access to PCR testing in Ethiopia is also sparse. Monitoring the epidemic by detecting symptomatic cases is, therefore, highly insensitive. In these circumstances, seroprevalence of anti-SARS-CoV-2 antibodies can provide a more accurate estimator of cumulative incidence. Undertaking community sero-sampling during the pandemic is difficult when travel and household access are constrained by control measures. Expectant mothers, however, are likely to continue to seek health services throughout the pandemic and they can be used as a continuously available proxy population to estimate the cumulative incidence among young adults [3-5]. In addition, serological surveillance is simple to implement at Antenatal Clinic (ANC) visits because anti-SARS-CoV-2 antibodies can be assayed in the residual blood volumes of routine samples collected for clinical screening for anaemia and maternal infectious diseases.

Planning and provision of health care during a major epidemic like COVID-19 pose substantial logistical and clinical challenges. Information on the shape of the epidemic curve is critical to inform public health responses. The dynamics of seroprevalence reflect the epidemic curve and can provide an estimate of the effective reproduction number. Seroprevalence also indicates the likelihood of approaching transmission control through population immunity. This study aimed to assess the trend in seroprevalence of anti-SARS-CoV-2 antibodies throughout the first year of the

For peer review only - http://bmjopen.bmj.com/site/about/guidelines.xhtml

epidemic by assaying anti-SARS-CoV-2 antibodies among pregnant women attending ANC at three different health facilities in the area around Harar, eastern Ethiopia.

Material and methods

Study area and period

The surveillance was conducted between April 1, 2020 and March 31, 2021 at Awoday Health Centre and Haramaya District Hospital, both in Haramaya District, and in Hiwot Fana Specialized Referral University Hospital in Harar. Hiwot Fana is the largest referral and teaching hospital in eastern Ethiopia and receives tertiary referrals from Harari region, East Oromia, Somali region, and Dire Dawa City. It is one of the ten regional centres designated by the Federal Ministry of Health to manage the COVID-19 epidemic. Haramaya Hospital was rapidly designated a COVID-19 treatment facility and women seeking ANC services were therefore referred to Awoday Health Centre after April 16, 2020. Ethiopia began to roll out COVID-19 vaccine in the first quarter of 2021; however, no doses were given in the study area during the period this analysis covers.

Study design, population and sample size

At the end of March 2020, we integrated Health facility-based surveillance into the routine clinical care of pregnant women at Hiwot Fana Hospital, Awoday Health Centre and Haramaya Hospital. The study population comprised 3306 pregnant women attending their first antenatal care in these three facilities during the surveillance period. Because we had fewer test kits available than there were samples available from the clinic, we selected a random sample for analysis stratified on month. Initially, we decided to select 144 samples per month; as the seroprevalence was very low in the first three months we reset the sample size to 80 per month from July onwards and increased

For peer review only - http://bmjopen.bmj.com/site/about/guidelines.xhtml

108 it to 160 per month in December once the seroprevalence reached 5%. In October, the number of samples collected was lower than the desired sample size and we therefore tested all of the samples (See Supplementary table).

A total of 78 women were excluded because they were not willing to provide a blood sample. Routine antenatal care includes serological screening for HIV, syphilis, and toxoplasma infection during pregnancy undertaken in two blood samples; the first blood sample is taken at 16 weeks' gestation or at the first ANC visit, if later.

Socio-demographic data and information on pregnancy, clinical symptoms of COVID-19 and co-morbidities was collected by trained nurses. COVID-19 symptoms were defined as at least one of cough, fever, headache or difficulty breathing. Data quality and completeness were checked daily.

Laboratory analyses

For the anti–SARS-CoV-2 antibodies test, residual blood samples from the routine ANC tests were transferred to a test-tube containing clot activator by trained medical laboratory technologists working in each health facility. The blood samples were allowed to clot and serum was separated by centrifugation at 3000 RPM for 10 minutes. Serum samples were stored at 2-8°C at each site and transported in cool boxes to Hararghe Health Research Laboratory where they were stored at -80°C.

Samples were tested using WANTAI® SARS-CoV-2 Ab Rapid Test. The test is a lateral flow assay in a cassette format designed for the qualitative detection of total antibodies to SARS-CoV-2 in human serum. The receptor-binding domain of the SARS-CoV-2 spike protein is bound at the

For peer review only

For peer review only - http://bmjopen.bmj.com/site/about/guidelines.xhtml

Test Zone (T) and antibodies are bound at the Control Zone (C) of the cassette. The test has a sensitivity of 100% and specificity of 98.8% under validation performed by the manufacturer 6; independent validation of the test found a sensitivity of 89% 7. All the stored serum samples, tests reagents and cassettes were brought to room temperature (15-30°C) thirty minutes before performing the test and checked for defects. Then, a 10µl of serum specimen and two drops of diluent buffer were added into the specimen window. Results were read and interpreted as reactive/positive (Red line on C and T) or non-reactive/negative (Red line on C) after 15-20 minutes according to manufacturer's instruction 6 7. Serum samples taken ≥14 days after a positive PCR test from COVID-19 infected individuals were used as positive control. Samples were tested in batches of 50-60 by a single operator. Assays without a valid reaction on the control line were rejected and the assay repeated on a new kit.

Patient and Public Involvement statement

Because the surveillance was set up urgently at the beginning of the pandemic we were not able to involve participants or the public in the design or set-up and because it was designed as an anonymous surveillance we were not able to provide individual feedback of the results to the participants. We have provided feedback of these high-level results through our existing community engagement exercises, including local radio programmes, meetings with local leaders and communication through health workers for onward dissemination.

Statistical analysis

We used STATA version 16.0 for statistical management and analysis. We selected a random sample of participants each month using the runiform function. We estimated unadjusted seroprevalence of SARS-CoV-2 IgG antibody with a 95% confidence interval (CI). We did not

For peer review only - http://bmjopen.bmj.com/site/about/guidelines.xhtml

150 make adjustment for the test performance characteristics because the manufacturer's validation assay, found very high sensitivity and specificity. We examined the univariate association between individual characteristics and seropositivity using Chi-square test and multivariable associations using binomial regression. The trend in seropositivity with time was tested with a Chi-square test for trend and in the multivariable model. Data used in the analysis is available at dataverse.harvard.edu 8.

156 **Ethical consideration**

157 The surveillance was confined to residual clinical blood sample testing and anonymized data were collected using checklists to extract data from ANC cards. Bar codes, representing an anonymous unique identity number, were used to link extracted clinical and demographic data with test sample results. The exercise was conducted as part of a public health surveillance, with the approval of the directors of each of the three health facilities, and the results were made available to health facilities, the Regional Health Bureau (Harari and Oromia) and the Ethiopian Public Health Institute (EPHI). All ANC attendees were informed that the clinic was participating in an anonymous surveillance and mothers were made aware that the residual volumes of their blood samples would be made available to the surveillance laboratory. Written individual informed consent was not obtained. The surveillance exercise was approved by the Institutional Health Research Ethical Review Committee of the College of Health and Medical Sciences, Haramaya University, Ethiopia with clearance number 123/2021.

For peer review only

For peer review only - http://bmjopen.bmj.com/site/about/guidelines.xhtml

Results

Demographic characteristics of the study participants

Between April 1, 2020 and March 31, 2021 there were 3,313 first visits to the antenatal clinics; 1,532 (46.24%) at Hiwot Fana Hospital, 1781 (53.75%) at Awoday Health Centre and Haramaya Hospital. At these, we interviewed and collected blood samples from 3,312 women. We tested a random sample of 1,447 blood specimens (Table 1); 752 (52%) were from Haramaya District (Awoday Health Centre and Haramaya Hospital) and 695 (48%) were from Hiwot Fana Hospital; 984 (68%) were urban residents.

Among the population sample tested, the mean (SD) age was 23.9 (4.7) years and ages ranged from 15 to 45 years. The mean (SD) number of children per mother was 1.5 (1.8). The median (IQR) gestational age at the first antenatal visit was 20 (13-28) weeks. Only 51 (3.5%) had COVID-19 symptoms at the time of sampling and 8 (<1%) had a history of comorbidity, given as chronic liver, renal, cardiovascular or 'other' disease. Respiratory diseases, chronic neurological disease, diabetes mellitus, and cancer were not reported by any participant.

Seroprevalence of SARS-CoV-2 antibodies

Of 1,447 samples tested, 83 (5.7%, 95% CI 4.6, 7.0%) were positive for anti-SARS-CoV-2 antibodies. The first seropositive sample was identified on June 11, 2020, and seroprevalence rose progressively thereafter, with the exception of March 2021, where it dropped sharply (chi-square for trend for the whole year, p=0.003; Figure 1).

For peer review only

Seroprevalence also varied significantly by trimester of pregnancy and co-morbidity but not by clinic, residence or COVID-19 symptoms (Table 2). Given the linear growth in seroprevalence (Figure 1) and better model fit based on Bayesian information criterion, we modelled prevalence associations as risk differences rather than risk ratios. In a multivariable binomial regression model, the prevalence difference was -3.2% (95% CI -6.7, -0.4%) and -3.0% (95% CI -6.8,-0.8%) among women in their second and third trimesters, respectively, compared with those in the first trimester and the prevalence difference was 0.75% (95% CI 0.31, 1.20%) per month of calendar time.

Discussion

The study provides a simple description of the dynamic of SARS-CoV-2 epidemic in an area where reliable data are extremely rare. In a population of attendees at antenatal clinics in three sites in eastern Ethiopia, antibodies against SARS-CoV-2 first appeared in June 2020 and seroprevalence rose steadily month on month reaching approximately 10% at the beginning of 2021. Although the point estimate for March 2021 is substantially lower, the data as a whole evince a strong linear trend and this single estimate is most likely to have deviated from the general direction by chance. If these results are reliable, they indicate that the epidemic is progressing here at a considerably lower rate than in other settings in East Africa and that the greater majority of the population remains uninfected, suggesting that the epidemic is still at an early stage.

The principal limitations of the study are the potential generalisability of the population under surveillance and the validity of the serological assay employed. Pregnant women have been used as an indicator population in prior pandemics, including HIV [9], but also for SARS-CoV-2, both in

For peer review only

For peer review only - http://bmjopen.bmj.com/site/about/guidelines.xhtml

high-income settings 4 5 10-14 and low- and middle-income settings, including in neighbouring Kenya 3. The principal advantage of sampling pregnant women is that they remain one of the few patient groups for whom health services cannot be postponed until after the pandemic has passed. They are permitted and encouraged to attend even in the face of social and movement restrictions, and so provide a consistent and reliable sampling group. The principal limitation of this group is their restriction on age and sex, however, in most settings, including other East African countries, seroprevalence does not vary significantly by sex and the cumulative incidence in women is likely to represent the infection history of both sexes 15-20. Similarly, in most settings young adults are the group most likely to be infected by SARS-CoV-2 and so the seroprevalence estimates here are likely to represent the highest risk in the whole population; other age groups, particularly children and the elderly, are likely to have lower seroprevalence 17 21.

The World Health Organization has deprecated the use of rapid tests for SARS-CoV-2 antibodies for individual diagnosis but recognises their potential value in research 22. WHO has also recommended and endorsed quantitative analysis of IgG antibodies using ELISA and has distributed the WANTAI ELISA kit to countries undertaking serosurveillance. Reliance on ELISA, however, limits the range of settings in which serosurveillance can be undertaken and lateral flow tests have been successfully employed for recurrent community-based nationwide surveys in the UK 23. When seroprevalence is low, as at the beginning of our study, an assay with imperfect specificity may detect more false positives than true positives. The specificity of the WANTAI rapid test has been estimated by the manufacturer at 98.8%. We assayed 80 samples each month in April and May 2020 without observing a single positive test, suggesting that the specificity is indeed very high. Even if the positive results identified in June included false

For peer review only - http://bmjopen.bmj.com/site/about/guidelines.xhtml

positives, the progressive rise in seropositivity with time is most unlikely to be influenced materially by a small fraction of false positive results.

The assay sensitivity may also be imperfect in detecting prior infection because the assay was originally calibrated against sera from symptomatic cases, who generally have higher antibody levels than asymptomatic individuals [24], and because pregnant women who were infected several months ago may have experienced waning of antibody levels and seroreversion [25-29]. In general, seroreversion is less problematic in assays that measure total immunoglobulin and in those that target spike antigens, compared to nucleocapsid antigens [27 30], so problem of waning in this study is unlikely to be substantial. Furthermore, if sensitivity is unlikely to decline over time, imperfect sensitivity would not affect the shape of the rising seroprevalence line, though it would underestimate the gradient. If, as estimated in one validation study, the WANTAI rapid test has a sensitivity of only 89% [7], adjustment for test-performance characteristics would elevate our reported seroprevalence results by a factor of 1.12.

For peer review only

The results are in contrast to most other settings studied which record a sharp take-off in seroprevalence once transmission begins, often rising quickly to high levels. In Kenya for example, women attending an ANC clinic in Kilifi had seroprevalence of 0%, 2% and 11% in consecutive months September-November 2021; those attending ANC clinic in Nairobi had a seroprevalence of 50% in August 2020 [3]. The pattern illustrated in eastern Ethiopia is more indicative of a gradually spreading epidemic curve suggesting an effective reproduction number much closer to 1.

For peer review only - http://bmjopen.bmj.com/site/about/guidelines.xhtml

In Juba, South Sudan, seroprevalence was 22% in a household survey in August-September 2020 20; in Kenya, a national estimate for seroprevalence, based on testing blood transfusion donors, was 4.3% in May 2020 18 and 9.1% two months later 15. Health Care Workers in Nairobi, Kenya, had a seroprevalence of 44% in August 2020; those in two rural hospitals had seroprevalence of 12-13% in November 2020 15. Finally, in Addis Ababa seroprevalence, estimated in May 2020, was 3.0% 31. Although all these studies used different laboratory assays and varied statistical adjustments, collectively, they suggest that transmission in eastern Ethiopia began later than in much of the rest of the region, including the state capital, and has progressed more slowly.

Conclusion

In summary, if seroprevalence is a reliable indicator of cumulative incidence, SARS-CoV-2 infection is spreading slowly but steadily in eastern Ethiopia. This contrasts sharply with the recurrent waves of PCR-positive infections apparent in the national surveillance system. One year after the start of the epidemic, approximately 10% of women attending antenatal clinics are seropositive implying that the COVID-19 epidemic is still at an early stage in eastern Ethiopia.

[**Dataset**] [8] Assefa N, Demissie L, Teklemarriam Z, Oundo J, Madrid L, Dessie Y, et al. *Data from*: Seroprevalence of anti–SARS-CoV-2 antibodies in women attending antenatal care in eastern Ethiopia: V1 ed: Harvard Dataverse; June 9, 2021. doi:10.7910/DVN/XIWCXN

For peer review only - http://bmjopen.bmj.com/site/about/guidelines.xhtml

References

1. Abdela SG, Abegaz SH, Demsiss W, et al. Clinical Profile and Treatment of COVID-19 Patients: Experiences from an Ethiopian Treatment Center. *Am J Trop Med Hyg* 2020 doi: 10.4269/ajtmh.20-1356 [published Online First: 2021/01/01]
2. Abraha HE, Gessesse Z, Gebrecherkos T, et al. Clinical features and risk factors associated with morbidity and mortality among patients with COVID-19 in northern Ethiopia. *Int J Infect Dis* 2021;105:776-83. doi: 10.1016/j.ijid.2021.03.037 [published Online First: 2021/03/21]
3. Lucinde R, Mugo D, Bottomley C, et al. Sero-surveillance for IgG to SARS-CoV-2 at antenatal care clinics in two Kenyan referral hospitals. *medRxiv* 2021:2021.02.05.21250735. doi: 10.1101/2021.02.05.21250735
4. Lumley SF, Eyre DW, McNaughton AL, et al. SARS-CoV-2 antibody prevalence, titres and neutralising activity in an antenatal cohort, United Kingdom, 14 April to 15 June 2020. *Euro Surveill* 2020;25(42) doi: 10.2807/1560-7917.ES.2020.25.41.2001721 [published Online First: 2020/10/24]
5. Villalaín C, Herraiz I, Luczkowiak J, et al. Seroprevalence analysis of SARS-CoV-2 in pregnant women along the first pandemic outbreak and perinatal outcome. *PLoS ONE* 2020;15(11):e0243029. doi: 10.1371/journal.pone.0243029
6. The WANTAI SARS-CoV-2 Ab Rapid Test. Beijing Wantai Biological Pharmacy Enterprise Co., Ltd. No.31 Kexueyuan Road, Changping District, Beijing 102206, China. 2020 [Available from: https://www.fda.gov/media/140030/download accessed 29-04-2021 2021.
7. Weidner L, Gänsdorfer S, Unterweger S, et al. Quantification of SARS-CoV-2 antibodies with eight commercially available immunoassays. *Journal of Clinical Virology* 2020;129:104540. doi: https://doi.org/10.1016/j.jcv.2020.104540
[**Dataset**] 8. Assefa N, Demissie L, Teklemarriam Z, et al. *Data from*: Seroprevalence of anti–SARS-CoV-2 antibodies in women attending antenatal care in eastern Ethiopia: doi:10.7910/DVN/XIWCXN. V1 ed: Harvard Dataverse-June 9, 2021.
9. Kigadye RM, Klokke A, Nicoll A, et al. Sentinel surveillance for HIV-1 among pregnant women in a developing country: 3 years' experience and comparison with a population serosurvey. *AIDS* 1993;7(6):849-55. doi: 10.1097/00002030-199306000-00014 [published Online First: 1993/06/01]
10. Cosma S, Borella F, Carosso A, et al. The "scar" of a pandemic: Cumulative incidence of COVID-19 during the first trimester of pregnancy. *Journal of medical virology* 2021;93(1):537-40. doi: 10.1002/jmv.26267 [published Online First: 2020/07/08]
11. Crovetto F, Crispi F, Llurba E, et al. Seroprevalence and presentation of SARS-CoV-2 in pregnancy. *Lancet* 2020;396(10250):530-31. doi: 10.1016/S0140-6736(20)31714-1 [published Online First: 2020/08/11]
12. Flannery DD, Gouma S, Dhudasia MB, et al. SARS-CoV-2 seroprevalence among parturient women in Philadelphia. *Sci Immunol* 2020;5(49) doi: 10.1126/sciimmunol.abd5709 [published Online First: 2020/07/31]
13. Haizler-Cohen L, Davidov A, Blitz MJ, et al. Severe acute respiratory syndrome coronavirus 2 antibodies in pregnant women admitted to labor and delivery units. *Am J Obstet Gynecol* 2021;224(1):112-14. doi: 10.1016/j.ajog.2020.09.022 [published Online First: 2020/09/25]

For peer review only - http://bmjopen.bmj.com/site/about/guidelines.xhtml

14. Mattern J, Vauloup-Fellous C, Zakaria H, et al. Post lockdown COVID-19 seroprevalence and circulation at the time of delivery, France. *PLoS ONE* 2020;15(10):e0240782. doi: 10.1371/journal.pone.0240782 [published Online First: 2020/10/16]
15. Adetifa IMO, Uyoga S, Gitonga JN, et al. Temporal trends of SARS-CoV-2 seroprevalence during the first wave of the COVID-19 epidemic in Kenya. *Nature Communications* 2021;12(3966) doi: 10.1038/s41467-021-24062-3
16. Etyang AO, Lucinde R, Karanja H, et al. Seroprevalence of Antibodies to SARS-CoV-2 among Health Care Workers in Kenya. *Clin Infect Dis* 2021 doi: 10.1093/cid/ciab346 [published Online First: 2021/04/25]
17. Pollan M, Perez-Gomez B, Pastor-Barriuso R, et al. Prevalence of SARS-CoV-2 in Spain (ENE-COVID): a nationwide, population-based seroepidemiological study. *Lancet* 2020;396(10250):535-44. doi: 10.1016/S0140-6736(20)31483-5 [published Online First: 2020/07/10]
18. Uyoga S, Adetifa IMO, Karanja HK, et al. Seroprevalence of anti-SARS-CoV-2 IgG antibodies in Kenyan blood donors. *Science* 2021;371(6524):79-82. doi: 10.1126/science.abe1916 [published Online First: 2020/11/13]
19. Uyoga S, Adetifa IMO, Otiende M, et al. Prevalence of SARS-CoV-2 Antibodies From a National Serosurveillance of Kenyan Blood Donors, January-March 2021. *Jama* 2021 doi: 10.1001/jama.2021.15265 [published Online First: 2021/09/03]
20. Wiens KE, Mawien PN, Rumunu J, et al. Seroprevalence of anti-SARS-CoV-2 IgG antibodies in Juba, South Sudan: a population-based study. *medRxiv* 2021 doi: 10.1101/2021.03.08.21253009 [published Online First: 2021/03/25]
21. Murhekar MV, Bhatnagar T, Selvaraju S, et al. SARS-CoV-2 antibody seroprevalence in India, August-September, 2020: findings from the second nationwide household serosurvey. *The Lancet Global health* 2021;9(3):e257-e66. doi: 10.1016/S2214-109X(20)30544-1 [published Online First: 2021/01/31]
22. World Health Organization. Advice on the use of point-of-care immunodiagnostic tests for COVID-19, 2020:https://www.who.int/news-room/commentaries/detail/advice-on-the-use-of-point-of-care-immunodiagnostic-tests-for-covid-19.
23. Moshe M, Daunt A, Flower B, et al. SARS-CoV-2 lateral flow assays for possible use in national covid-19 seroprevalence surveys (React 2): diagnostic accuracy study. *BMJ (Clinical research ed)* 2021;372:n423. doi: 10.1136/bmj.n423 [published Online First: 2021/03/04]
24. Takahashi S, Greenhouse B, Rodriguez-Barraquer I. Are Seroprevalence Estimates for Severe Acute Respiratory Syndrome Coronavirus 2 Biased? *J Infect Dis* 2020;222(11):1772-75. doi: 10.1093/infdis/jiaa523 [published Online First: 2020/08/29]
25. Ibarrondo FJ, Fulcher JA, Goodman-Meza D, et al. Rapid Decay of Anti-SARS-CoV-2 Antibodies in Persons with Mild Covid-19. *The New England journal of medicine* 2020;383(11):1085-87. doi: 10.1056/NEJMc2025179 [published Online First: 2020/07/25]
26. Long QX, Tang XJ, Shi QL, et al. Clinical and immunological assessment of asymptomatic SARS-CoV-2 infections. *Nat Med* 2020;26(8):1200-04. doi: 10.1038/s41591-020-0965-6 [published Online First: 2020/06/20]
27. Lumley SF, Wei J, O'Donnell D, et al. The duration, dynamics and determinants of SARS-CoV-2 antibody responses in individual healthcare workers. *Clin Infect Dis* 2021 doi: 10.1093/cid/ciab004 [published Online First: 2021/01/06]

For peer review only - http://bmjopen.bmj.com/site/about/guidelines.xhtml

28. Muecksch F, Wise H, Batchelor B, et al. Longitudinal Serological Analysis and Neutralizing Antibody Levels in Coronavirus Disease 2019 Convalescent Patients. *J Infect Dis* 2021;223(3):389-98. doi: 10.1093/infdis/jiaa659 [published Online First: 2020/11/04]
29. Ward H, Cooke GS, Atchison C, et al. Prevalence of antibody positivity to SARS-CoV-2 following the first peak of infection in England: Serial cross-sectional studies of 365,000 adults. *Lancet Reg Health Eur* 2021;4:100098. doi: 10.1016/j.lanepe.2021.100098 [published Online First: 2021/05/11]
30. Post N, Eddy D, Huntley C, et al. Antibody response to SARS-CoV-2 infection in humans: A systematic review. *PLoS ONE* 2020;15(12):e0244126. doi: 10.1371/journal.pone.0244126 [published Online First: 2021/01/01]
31. Kempen JH, Abashawl A, Suga HK, et al. SARS-CoV-2 Serosurvey in Addis Ababa, Ethiopia. *Am J Trop Med Hyg* 2020;103(5):2022-23. doi: 10.4269/ajtmh.20-0816 [published Online First: 2020/09/26]

Author contribution

NA and JAGS lead overall Surveillance. NA, JAGS, ZT and LDR develop concept of the Surveillance, analysed the data and wrote the manuscript. ZT provided microbiome data analysis, and interpretation. NA, JAGS, ZT, LDR, LM, JO and YD reviewed the manuscript and give critical feedback. All authors approved the submission of the manuscript to the journal.

Competing of interest

The authors declare no competing interests.

Funding:

Activities of the surveillance and Laboratory analysis was supported by Emory University as part of the CHAMPS network (www.champshealth.org). JAGS was funded by The Wellcome Trust through a Senior Research Fellowship (214320).

For peer review only - http://bmjopen.bmj.com/site/about/guidelines.xhtml

Data availability

Data is available in the following link and can be requested using the form in the link. https://dataverse.harvard.edu/dataset.xhtml?persistentId=doi:10.7910/DVN/XIWCXN.

Acknowledgements

Investigators would like to thank authorities of the health facilities for facilitating the surveillance and pregnant women for voluntarily participating in the study.

For peer review only

Figure titles/legends

Figure 1: Temporal trend of seroprevalence of anti–SARS-CoV-2 antibodies among pregnant women presenting for first antenatal care in 3 ANC facilities, eastern Ethiopia, between April 1, 2020 and March 31, 2021

For peer review only

For peer review only - http://bmjopen.bmj.com/site/about/guidelines.xhtml

Table 1. Characteristics of 1,447 pregnant mothers attending their first antenatal care at the two study clinics between April 2020 and March 2021 and sampled at random for the study.

**Characteristics**	**Haramaya District**		**Hiwot Fana Hospital**		**Total**	
	**n**	**%**	**n**	**%**	**n**	**%**
Age in years						
14-19	98	13.0	74	13.5	192	13.3
20-24	341	45.4	268	35.6	588	40.7
25-29	181	24.1	219	31.6	400	27.7
30-34	110	14.6	100	14.4	210	14.5
>=35	22	2.9	35	4.9	56	3.9
Residence						
Urban	577	76.8	407	58.7	984	68.1
Rural	174	23.2	287	41.4	461	31.9
Number of children						
None	298	40.0	254	36.8	552	38.4
1-5	422	56.6	401	58.1	823	57.3
6-10	26	3.5	35	5.1	61	4.3
Trimester of visit						
First	223	29.7	143	20.6	366	25.3
Second	416	55.3	242	34.8	658	45.5
Third	113	15.0	310	44.6	423	29.2
Comorbidities						
None	750	99.7	689	99.1	1,439	99.5
At least one*	2	0.3	6	0.9	8	0.6
COVID symptoms†						
No	721	96.4	668	96.5	1,389	96.5
Yes	27	3.6	24	3.5	51	3.5

* chronic liver, renal, cardiovascular or 'other' disease

† at least one of cough, fever, headache or difficulty breathing

For peer review only - http://bmjopen.bmj.com/site/about/guidelines.xhtml

For peer review only

Table 2. Seroprevalence of Anti-SARS-CoV-2 antibodies by participant characteristics.

**Characteristics**	**Tested N**	**Sero-positive n**	**Sero-prevalence %**	**Chi-square test p value**
Age in years				
14-19	192	7	3.7	0.19
20-24	588	42	7.1	
25-29	400	24	6.0	
30-34	210	9	4.3	
>=35	56	1	1.8	
ANC clinic				
Hiwot Fana Hospital	695	35	5.0	0.260
Haramaya Hospital	19	0	0.0	
Awoday Health Centre	733	48	6.6	
Residence				
Urban	984	57	5.8	0.910
Rural	461	26	5.6	
Number of children				
None	520	32	5.8	0.370
1-5	778	45	5.5	
6-10	55	6	9.8	
Trimester of visit				
First	366	31	8.5	0.034
Second	658	32	4.9	
Third	423	20	4.8	
Comorbidities				
None	1,439	81	5.6	0.019
At least one*	8	2	25.0	
COVID symptoms†				
No	1,389	82	5.9	0.240
Yes	51	1	2.0	

* chronic liver, renal, cardiovascular or 'other' disease

† at least one of cough, fever, headache or difficulty breathing

For peer review only - http://bmjopen.bmj.com/site/about/guidelines.xhtml

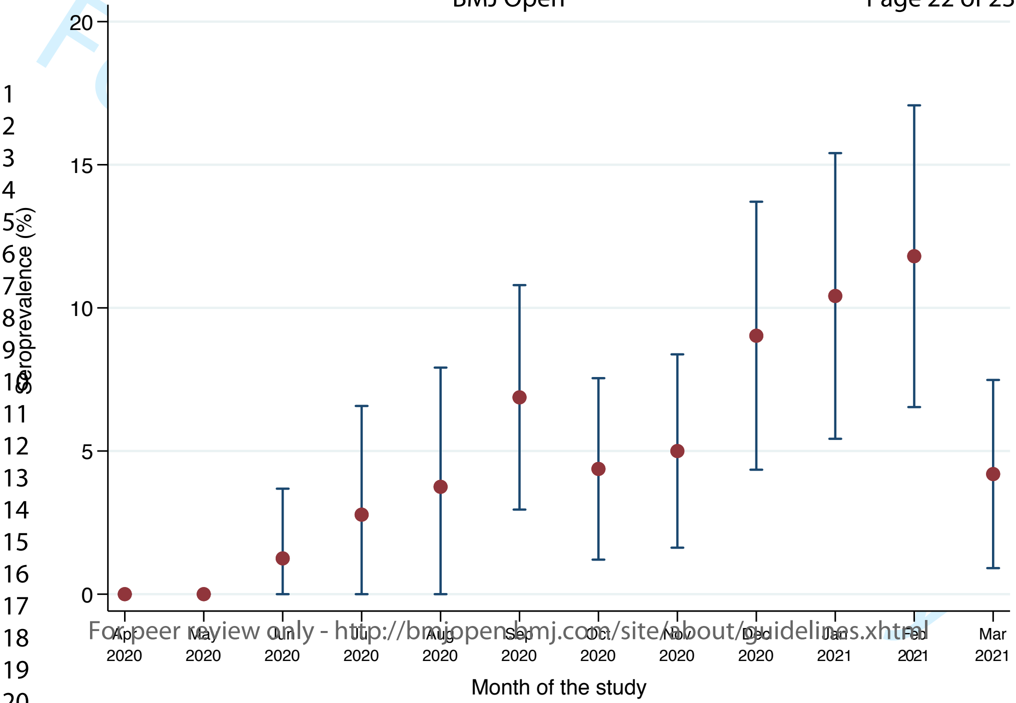

For peer review only - http://bmjopen.bmj.com/site/about/guidelines.xhtml

Seroprevalence of anti–SARS-CoV-2 antibodies in women attending antenatal care in eastern Ethiopia

STROBE Statement—Checklist of items that should be included in reports of ***cross-sectional studies***

	Item No	Recommendation	Page No
**Title and abstract**	1	(*a*) Indicate the study's design with a commonly used term in the title or the abstract	1
		(*b*) Provide in the abstract an informative and balanced summary of what was done and what was found	1
**Introduction**			
Background/rationale	2	Explain the scientific background and rationale for the investigation being reported	4
Objectives	3	State specific objectives, including any prespecified hypotheses	4-5
**Methods**			
Study design	4	Present key elements of study design early in the paper	5
Setting	5	Describe the setting, locations, and relevant dates, including periods of recruitment, exposure, follow-up, and data collection	5
Participants	6	(*a*) Give the eligibility criteria, and the sources and methods of selection of participants	5-6
Variables	7	Clearly define all outcomes, exposures, predictors, potential confounders, and effect modifiers. Give diagnostic criteria, if applicable	6
Data sources/ measurement	8*	For each variable of interest, give sources of data and details of methods of assessment (measurement). Describe comparability of assessment methods if there is more than one group	7
Bias	9	Describe any efforts to address potential sources of bias	7
Study size	10	Explain how the study size was arrived at	5
Quantitative variables	11	Explain how quantitative variables were handled in the analyses. If applicable, describe which groupings were chosen and why	6-7
Statistical methods	12	(*a*) Describe all statistical methods, including those used to control for confounding	7
		(*b*) Describe any methods used to examine subgroups and interactions	7
		(*c*) Explain how missing data were addressed	7
		(*d*) If applicable, describe analytical methods taking account of sampling strategy	7
		(*e*) Describe any sensitivity analyses	7
**Results**			
Participants	13*	(a) Report numbers of individuals at each stage of study—eg numbers potentially eligible, examined for eligibility, confirmed eligible, included in the study, completing follow-up, and analysed	8
		(b) Give reasons for non-participation at each stage	8
		(c) Consider use of a flow diagram	-
Descriptive data	14*	(a) Give characteristics of study participants (eg demographic, clinical, social) and information on exposures and potential confounders	8-10
		(b) Indicate number of participants with missing data for each variable of interest	8
Outcome data	15*	Report numbers of outcome events or summary measures	8-9

For peer review only - http://bmjopen.bmj.com/site/about/guidelines.xhtml

Seroprevalence of anti–SARS-CoV-2 antibodies in women attending antenatal care in eastern Ethiopia

Main results	16	(*a*) Give unadjusted estimates and, if applicable, confounder-adjusted estimates and their precision (eg, 95% confidence interval). Make clear which confounders were adjusted for and why they were included	8-9
		(*b*) Report category boundaries when continuous variables were categorized	9
		(*c*) If relevant, consider translating estimates of relative risk into absolute risk for a meaningful time period	-
Other analyses	17	Report other analyses done—eg analyses of subgroups and interactions, and sensitivity analyses	9
**Discussion**			
Key results	18	Summarise key results with reference to study objectives	9-10
Limitations	19	Discuss limitations of the study, taking into account sources of potential bias or imprecision. Discuss both direction and magnitude of any potential bias	10
Interpretation	20	Give a cautious overall interpretation of results considering objectives, limitations, multiplicity of analyses, results from similar studies, and other relevant evidence	10-12
Generalisability	21	Discuss the generalisability (external validity) of the study results	11-12
**Other information**			
Funding	22	Give the source of funding and the role of the funders for the present study and, if applicable, for the original study on which the present article is based	16

*Give information separately for exposed and unexposed groups.

Note: An Explanation and Elaboration article discusses each checklist item and gives methodological background and published examples of transparent reporting. The STROBE checklist is best used in conjunction with this article (freely available on the Web sites of PLoS Medicine at http://www.plosmedicine.org/, Annals of Internal Medicine at http://www.annals.org/, and Epidemiology at http://www.epidem.com/). Information on the STROBE Initiative is available at www.strobe-statement.org.

For peer review only

For peer review only - http://bmjopen.bmj.com/site/about/guidelines.xhtml
